# Opioid Prescribing Patterns After Imposition of Setting-Specific Limits on Prescription Duration

**DOI:** 10.1001/jamahealthforum.2023.4731

**Published:** 2024-01-19

**Authors:** Lindsay D. Allen, Robin A. Pollini, Richard Vaglienti, David Powell

**Affiliations:** 1 Department of Emergency Medicine, Feinberg School of Medicine, Northwestern University, Chicago, Illinois; 2 Department of Epidemiology and Biostatistics, School of Public Health, West Virginia University, Morgantown; 3 Center for Integrative Pain Management, West Virginia University, Morgantown; 4 RAND Corporation, Arlington, Virginia

## Abstract

**Importance:**

Despite their widespread adoption across the US, policies imposing one-size-fits-all limits on the duration of prescriptions for opioids have shown modest and mixed implications for prescribing.

**Objective:**

To assess whether a prescription duration limit policy tailored to different clinical settings was associated with shorter opioid prescription lengths.

**Design, Setting, and Participants:**

This cross-sectional study examined changes in opioid prescribing patterns for opioid-naive Medicaid enrollees aged 12 to 64 years before and after implementation of a statewide prescription duration limit policy in West Virginia in June 2018. Patients with cancer or Medicare coverage were excluded. The policy assigned a 7-day duration limit to opioid prescriptions for adults treated in outpatient hospital- or office-based practices, a 4-day limit for adults treated in emergency departments, and a 3-day limit for pediatric patients younger than 18 years regardless of clinical setting. Data were examined from January 1, 2017, through September 30, 2019, and data were analyzed from June 12 to October 30, 2023.

**Main Outcomes and Measures:**

Whether a patient’s initial opioid prescription was longer in days than the June 2018 policy limit for a given care setting before and after policy implementation. Interrupted time series models were used to calculate the association between the policy’s implementation and outcomes.

**Results:**

The analytic sample included 44 703 Medicaid enrollees (27 957 patients [62.5%] before policy implementation and 16 746 patients [37.5%] after policy implementation; mean [SD] age, 33.9 [13.4] years; 27 461 females [61.4%]). Among adults treated in outpatient hospital- or office-based settings, the duration limit policy was associated with a decrease of 8.83 (95% CI, −10.43 to −7.23) percentage points (*P* < .001), or a 56.8% relative reduction, in the proportion of prescriptions exceeding the 7-day limit. In the emergency department setting, the policy was associated with a decrease of 7.03 (95% CI, −10.38 to −3.68) percentage points (*P* < .001), a 37.5% relative reduction, in the proportion of prescriptions exceeding the 4-day limit. The proportion of pediatric opioid prescriptions longer than the 3-day limit decreased by 12.80 (95% CI, −17.31 to −8.37) percentage points (*P* < .001), a 26.5% relative reduction, after the policy’s implementation.

**Conclusions and Relevance:**

Results of this cross-sectional study suggest that opioid prescription duration limits tailored to different clinical settings are associated with reduced length of prescriptions for opioid-naive patients. Additional research is needed to evaluate whether these limits are associated with reductions in the incidence of opioid use disorder or with unintended consequences, such as shifts to illicit opioids.

## Key Points

**Question:**

Do tailored limits on the duration of opioid prescriptions reduce the length of prescriptions for opioid-naive patients?

**Findings:**

This cross-sectional study of 44 703 Medicaid enrollees who received an opioid prescription from 2017 to 2019 before or after implementation of a policy that limited the duration of opioid prescriptions found that implementation was associated with relative reductions of 56.8% for adults in outpatient practices, 37.5% for adults in emergency departments, and 26.5% for pediatric patients in any setting in the proportion of opioid prescriptions exceeding the duration limits.

**Meaning:**

The findings of this cross-sectional study suggest that policies tailored to specific clinical settings may be associated with reduced lengths of opioid prescriptions.

## Teaser

This cross-sectional study examines changes in the duration of opioid prescriptions before and after implementation of setting-specific prescription limits for opioid-naive patients.

## Introduction

The opioid crisis continues in the US. Exacerbated by the COVID-19 pandemic, the annual opioid-related death count rose to over 75 000 deaths in 2021.^1^ To minimize patient exposure to opioids, many states have passed laws restricting the number of days’ supply for first-time opioid prescriptions.^2^ The rationale for these limits is that shorter prescription duration may be less likely to result in downstream opioid dependence and may reduce diversion of unused medication.

However, evidence on prescribing limits has shown modest and mixed results.^3^ While a 7-day supply limit imposed in Massachusetts was associated with both fewer days’ supply and prescriptions for longer than 7 days, the same limit imposed in Connecticut did not result in any changes to prescription duration.^3,4^ Another study found a small decline in the duration of opioid prescriptions in Connecticut and Massachusetts after implementation of opioid prescription limits but not in New York.^5^ States that implemented opioid prescription limits in 2016 and 2017 did not have substantially greater decreases in opioid distribution volume compared with states without these laws.^6^ Across 13 states that implemented opioid prescribing laws (including prescription duration limits, pill mill laws, and prescription drug monitoring program policies), changes in prescribing attributable to the laws were consistently small in magnitude and not statistically significant compared with baseline data.^7^ Prescription duration limits among Medicare enrollees decreased total days’ supplied by 1.7 days per year (not per prescription).^8^

One possible reason for these modest and varied changes in opioid prescription duration is that the limits do not generally restrict prescriptions to shorter durations than clinicians would normally prescribe.^9^ The opioid duration limits across the US were not based on direct empirical evidence but rather arose after the Centers for Disease Control and Prevention (CDC) published its 2016 Opioid Prescribing Guidelines.^10^ Later, the CDC commented^11^ that state policies purportedly derived from the guideline were inconsistent with the guidelines’ recommendations, specifically calling out the application of inflexible prescription duration limits. As of 2019, 24 states imposed a prescription duration limit of 7 days, and only 5 states imposed statewide limits shorter than 7 days (eTable 1 in Supplement 1).^12^ Yet in December 2017, the mean duration of initial opioid prescriptions was 5.1 days among commercially insured patients.^3,13^ Because most opioid prescription lengths are already close to or below the mandated limit, many clinicians will not need to change their prescribing practices to remain in compliance with new state laws. Policies better tailored to current prescribing habits might be more effective in curbing prescription duration, but to our knowledge, this type of approach has not been empirically tested.

In this study, we examined the implications of a unique policy in West Virginia, a state at the epicenter of the opioid crisis. In June 2018, West Virginia implemented the Opioid Reduction Act, which assigned different days’ supply limits for opioid prescriptions in different clinical settings regardless of insurance type.^14^ We leveraged this natural experiment to contribute to the opioid policy literature in several ways. First, we tested for changes in opioid prescribing around the thresholds assigned for different clinical settings: 7 days for adults treated in outpatient hospital- or office-based practices, 4 days for adults treated in emergency departments (EDs), and 3 days for pediatric patients (younger than 18 years) treated in any setting.^14^ This allowed us to discern whether tailored policies (ie, fewer days’ supply limits in settings where opioid prescriptions already tend to be shorter) are associated with changes in prescribing patterns. Second, we studied opioid prescribing for pediatric patients, who had a separate prescription limit that has not been independently studied in prior work on prescription limits. This is an important knowledge gap given recently documented increases in opioid-associated deaths in the pediatric population.^15^ Next, we tested whether prescribers wrote stronger or more frequent prescriptions to compensate for shorter prescription lengths, which may attenuate the intended impact of the policy. Thus far, evidence on whether this compensatory prescribing occurs has been mixed.^5,16^ Finally, we focused the present study on Medicaid enrollees, who have been disproportionately affected by the opioid epidemic.^17,18^ Medicaid beneficiaries are prescribed more opioids than nonenrollees, largely reflecting that patients who qualify for Medicaid (whether through disability or not) are more likely to have chronic conditions and comorbidities that require pain relief.^19^ Among Medicaid enrollees, opioid prescription duration limits have been associated with a fewer number of opioid prescriptions, but their implications for prescription duration (ie, the policy’s target) has not been examined.^20^

## Methods

We used West Virginia Medicaid claims data from 2017 to 2019 for enrollees aged 12 to 64 years, excluding those with active cancer (who were exempted from the policy) or Medicare coverage. We identified opioid-naive patients using a 1-year lookback period and then selected those who had a health care visit that coincided with an initial Schedule II opioid prescription (ie, those targeted by the policy, such as oxycodone) within 1 day of the visit. Demographic data included age and sex. The primary outcome was whether an opioid prescription was longer in days than the June 2018 policy limit given the age of the patient and the care setting (eg, if a visit was made by an adult and took place in an ED, then the outcome is whether the resulting prescription was for >4 days). Other outcomes included prescription length in days, daily morphine milligram equivalents (MMEs) per prescription, the proportion of high-dose prescriptions (ie, >90 daily MMEs), and the proportion of prescriptions that were followed by a second opioid prescription within 30 days. We studied these outcomes before and after the enactment of the West Virginia policy in June 2018, aggregating outcomes at the monthly level. The Institutional Review Board of Northwestern University approved this cross-sectional study with a waiver of informed consent because data were deidentified.

### Statistical Analysis

We plotted the unadjusted proportion of initial opioid prescriptions that were longer than the setting-specific duration limits established in June 2018, both before (preperiod) and after (postperiod) implementation of the policy. We included the proportion of initial opioid prescriptions above the limit duration even before the policy was implemented (ie, the preperiod) so that we could observe how this rate changed. We excluded the month of June 2018 from analysis because the policy was introduced in the middle of that month. To quantify the visual evidence, we used an interrupted time series, ordinary least squares model that estimated the association between the Opioid Reduction Act and opioid prescription outcomes. The model included a linear time variable (month since the start of the study), a time-since-the-intervention variable (months since the policy was implemented), and a dichotomous variable for whether a given monthly observation occurred after the policy was introduced. The coefficient on this variable represents the level shift in an outcome that was associated with the introduction of the policy. Data were analyzed from June 12 to October 30, 2023, using Stata 16 (StataCorp, LLC). A 2-sided *P* < .05 was considered statistically significant.

## Results

The analytic sample was composed of 44 703 Medicaid enrollees (27 957 [62.5%] in the preperiod and 16 746 [37.5%] in the postperiod) who received an initial prescription for an opioid medication from January 1, 2017 to September 30, 2019 (Table 1). The mean (SD) age of the enrollees was 33.9 (13.4) years; 27 461 (61.4%) were female and 17 242 (38.6%) were male.

**Table 1.** Characteristics of Medicaid Enrollees Receiving Initial Opioid Prescriptions in West Virginia, 2017 to 2019

Characteristic	Enrollees, No. (%)			*P* value^a^
	Overall (N = 44 703)	Preperiod (n = 27 957)	Postperiod (n = 16 746)	
Age, mean (SD), y	33.9 (13.4)	34.1 (12.7)	33.6 (14.3)	<.001
Sex				
Female	27 461 (61.4)	17 271 (61.8)	10 190 (60.9)	.05
Male	17 242 (38.6)	10 686 (38.2)	6556 (39.1)	

^a^ *P* values denote statistical differences between the preperiod and postperiod groups and were calculated using *t* tests.

The Figure shows the unadjusted proportion of opioid prescriptions that were longer than 7 days for adults treated in an outpatient hospital- or office-based setting, longer than 4 days for adults treated in the ED, and longer than 3 days among pediatric patients treated in any setting. While these proportions were declining in the preperiod, especially among pediatric patients, we observed a substantial decrease across all 3 settings after the policy was implemented in mid-June 2018. These reductions began in the first full month after the implementation of the policy and persisted to the end of the study period. For example, the share of prescriptions exceeding the limit among office-based patients was approximately 15% in the preperiod (Figure, A) but decreased to approximately 5% after the policy's implementation.

**Figure.**
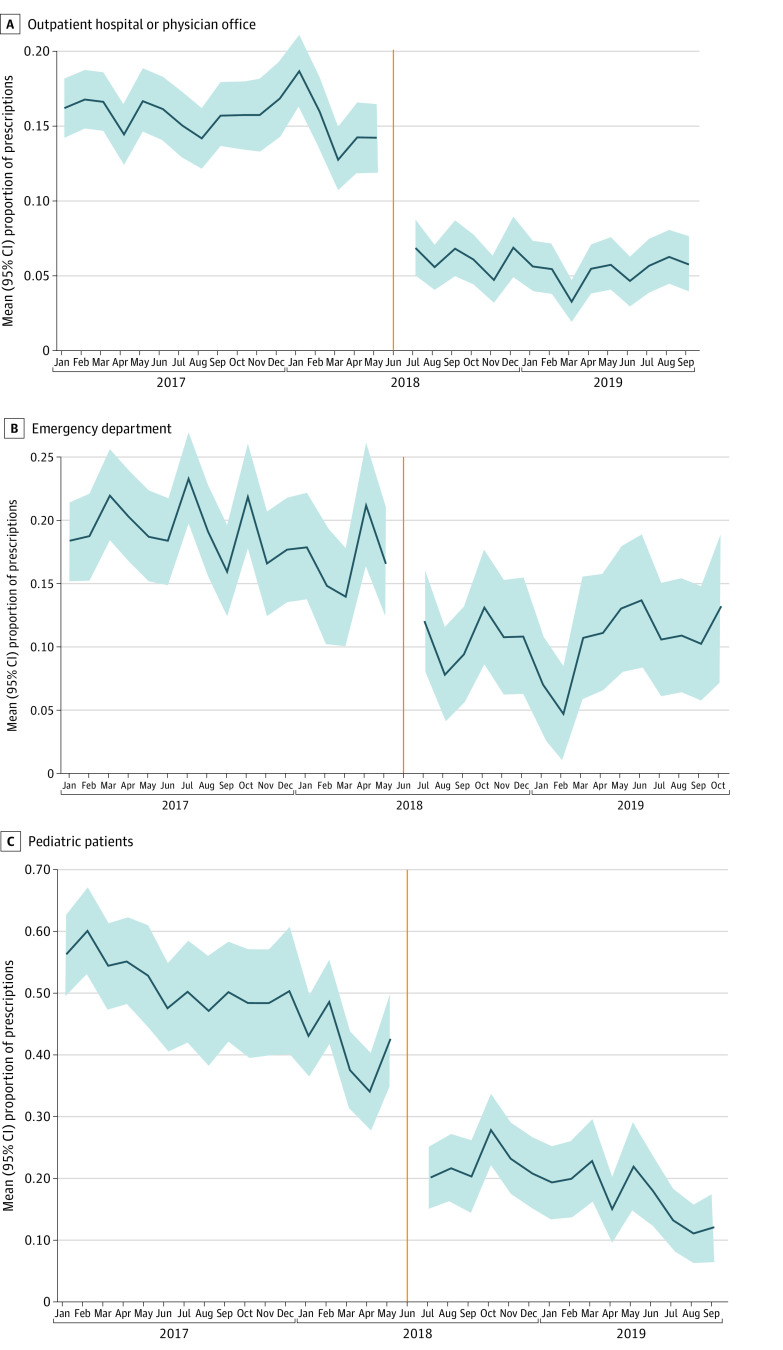
Proportion of Opioid Prescriptions Longer Than Duration Limits Before and After Duration Limits Were Imposed, 2017 to 2019 Unadjusted mean (95% CI) proportion of opioid prescriptions longer than the setting-specific duration limits imposed by the West Virginia Opioid Reduction Act of 2018, which set limits of 7 days for opioids prescribed to adults in an outpatient hospital- or office-based setting (A), 4 days for opioids prescribed to adults in the emergency department (B), and 3 days for opioids prescribed to pediatric patients in any setting. Vertical line indicates June 2018, when the opioid prescription limit policy was enacted.

Table 2 shows that in an outpatient hospital- or office-based setting, 15.56% of opioid prescriptions for adults exceeded the 7-day prescription limit in the preperiod; this proportion decreased by 8.83 (95% CI, −10.43 to −7.23) percentage points (*P* < .001), which corresponds to a 56.75% relative reduction after the policy’s implementation. Among adults treated in the ED, 18.75% of opioid prescriptions exceeded the 4-day limit in the preperiod, decreasing by 7.03 (95% CI, −10.38 to −3.68) percentage points (*P* < .001) in the postperiod, a relative decrease of 37.49%. Almost half (48.37%) of opioid prescriptions for pediatric patients were longer than the 3-day limit in the preperiod, decreasing by 12.80 (95% CI, −17.31 to −8.37) percentage points (*P* < .001) in the postperiod, a 26.46% relative reduction.

**Table 2.** Characteristics of Initial Opioid Prescriptions Among Medicaid Enrollees in West Virginia, 2017 to 2019

Characteristic	Prescribed for adults in outpatient hospital or physician office			Prescribed for adults in emergency department			Prescribed for pediatric patients		
	Preperiod mean, %	Level shift (95% CI), percentage points	Relative change, %^a^	Preperiod mean, %	Level shift (95% CI), percentage points	Relative change, %^a^	Preperiod mean, %	Level shift (95% CI), percentage points	Relative change, %^a^
Prescriptions over days’ limit	15.56	−8.83 (−10.43 to −7.23)	−56.75	18.75	−7.03 (−10.38 to −3.68)	−37.49	48.37	−12.80 (−17.31 to −8.37)	−26.46
No. of days supplied	5.44	−0.83 (−1.07 to −0.59)	−15.26	3.34	−0.10 (−0.28 to 0.08)	−2.99	4.13	−0.42 (−0.67 to −0.17)	−10.17
Daily MMEs	36.98	0.10 (−0.85 to 1.06)	0.27	29.39	0.42 (−0.94 to 1.77)	1.43	34.26	2.17 (0.77 to 3.57)	6.33
>90 MMEs	1.63	−1.07 (−1.66 to −0.48)	−65.64	0.68	−0.15 (−0.85 to 0.54)	−22.06	0.41	0 (−0.54 to 0.54)	0
Second prescription in 30 d	16.30	−1.91 (−3.70 to −0.13)	−11.72	18.67	−0.22 (−3.71 to 3.28)	−1.18	4.75	−0.65 (−2.60 to 1.30)	−13.68

Abbreviation: MMEs, milligram morphine equivalents.

^a^ Relative change was calculated from the preperiod mean, which was calculated from January 2017 to May 2018.

For adults treated in outpatient hospital- or office-based settings in whom the mean preperiod days of opioids supplied was 5.44 days, prescription length decreased by 0.83 days (95% CI, −1.07 to −0.59 days; *P* < .001), a relative decrease of 15.26% during the postperiod. For pediatric patients in whom the mean preperiod days’ supply of opioids was 4.13 days, prescription length decreased by 0.42 (95% CI, −0.67 to −0.17) days (*P* < .001), a relative decrease of 10.17% during the postperiod.

The remaining outcomes were designed as tests for compensatory prescribing (Table 2). Among pediatric patients, we found that the policy was associated with a slight increase of 2.17 (95% CI, 0.77 to 3.57) daily MMEs (*P* < .01), a relative increase of 6.33% over the preperiod mean of 34.26 MMEs. For adults treated in an outpatient hospital- or office-based setting, we observed a 1.07% decrease (*P* < .001) in the probability that a prescription was greater than 90 MMEs, a relative decrease of 65.64% from the preperiod mean of 1.63%. For adults treated in an outpatient hospital- or office-based setting, we also observed a decrease of 1.91 percentage points (95% CI, −3.70 to −0.13 percentage points; *P* = .04) in the probability that a patient received a second opioid prescription within 30 days of the first, a relative decrease of 11.72% from the preperiod mean of 16.30%. Full model results are available in eTable 2 in Supplement 1.

## Discussion

We examined changes in opioid prescribing patterns among Medicaid enrollees after the passage of legislation limiting opioid prescription durations in West Virginia in June 2018. The Opioid Reduction Act set stricter limits in settings where opioid prescriptions are traditionally shorter (ie, in the ED and among pediatric patients) and longer ones elsewhere (ie, in outpatient hospital- and office-based settings). Implementation of the law was associated with decreases in the proportion of opioid prescriptions longer than the respective day limits in each of the 3 settings and with decreases in the days’ supplied per opioid prescription in the adult outpatient setting and for pediatric patients.

Results of the present study contrast with those reported by Sedney et al,^21^ who found no effect of the same West Virginia policy change on mean days of opioids supplied per prescription. We believe there are 2 possible reasons for these different findings. First, the present study focused on the Medicaid population, who are prescribed opioids more frequently than commercially insured patients and may therefore be more likely to be affected by the new policy, while Sedney et al^21^ used prescription drug monitoring program data that includes both publicly and privately insured patients. Second, our study excluded patients with cancer, who were exempted from the West Virginia law. Almost half of patients with a new cancer diagnosis receive opioid prescriptions, and their inclusion in the study by Sedney et al^21^ might have biased those results toward the null, as this patient group is unlikely to be substantially affected by the policy.^22^

For adults, we did not find evidence that clinicians are writing stronger or more follow-up prescriptions as a way of compensating for shorter prescription durations. Instead, the policy was associated with reductions in high-dose and follow-up prescriptions in the adult outpatient setting. Two mechanisms may help explain these findings. First, the law may have heightened concerns about lengthy opioid use, discouraging clinicians’ willingness to write follow-up prescriptions. Second, longer initial prescriptions may have induced demand for subsequent prescriptions by building tolerance. By reducing prescription length, these subsequent prescriptions were not needed or requested.

Findings of the present study suggest a reduction in overall access to prescription opioids for Medicaid patients. It is not yet known how limiting opioid prescribing affects clinical outcomes. One study estimated that even substantial prevention of prescription opioid misuse would result in only a modest decrease of 3.8% to 5.3% in opioid-related overdose deaths.^23^ Against this small potential benefit, policymakers must weigh the likelihood of unintended consequences of duration limits. Reducing the supply of prescription opioids may increase the number of patients with uncontrolled pain, which has been shown to increase suicide risk.^24,25,26^ Furthermore, patients may turn to illicit, deadlier forms of opioids (eg, heroin or illegally manufactured fentanyl) if they are unable to obtain legally prescribed opioids; in turn, this transition may increase opioid-related morbidity and mortality.^27,28^ This phenomenon has been documented in a qualitative study of clinicians in West Virginia after the duration limit was introduced: opioid prescribers perceived that patients who were left without opioids transitioned to illicit substances, such as heroin.^27^ One factor mitigating these concerns is that duration limit policies tend to target opioid-naive patients with acute pain management needs, for whom these potential unintended consequences are less likely to be severe.

A key contribution of this study is the examination of the policy’s implications for opioid prescriptions written for pediatric patients. The pediatric population was the only setting in which the policy was associated with an increase in prescription strength. Furthermore, despite the substantial reduction in the proportion of opioid prescriptions that were over the duration limit after the policy was introduced, the rate of over-the-limit prescriptions remained (on average) at about 19% in the postperiod for pediatric patients. This finding is in contrast with the adult settings, where much smaller proportions of opioid prescriptions were over the limit after the policy was implemented. There are at least 2 potential reasons for this finding. First, the 3-day prescription length limit for pediatric patients represents one of the strictest limits in the country; this 3-day limit might represent the lower boundary on how far prescribers are willing to go when it comes to limiting pain treatment, regardless of whether the patient is a minor or an adult. Second, clinicians might be less willing to withhold potentially necessary pain medication from pediatric patients. One way to test this hypothesis would be by examining opioid prescription duration across pediatric and adult patients in a setting, such as Kentucky, where a 3-day duration limit has been applied to all patient groups.

### Limitations

A limitation of this study is that it did not control for other West Virginia–specific policy changes that might have occurred during the same time. Furthermore, we did not use data from a comparison state to control for secular trends that may have occurred across the study period. For these reasons, we did not make any claims about causality. We note, however, that no other policies directed at reducing the number of days’ supply of opioids were implemented in West Virginia in the same period, and we are not aware of any secular shifts that would have resulted in a sharp trend break in June 2018. We did not test for changes in the rate of new opioid prescriptions, a possible consequence of the policy that is outside the scope of this study. As a result, the characteristics we observed could pertain to prescriptions for patients whom clinicians deemed more clinically needful of pain management. If the policy also decreased the rate of new opioid prescriptions, this selection effect might bias our results toward the null.

## Conclusions

The results of this cross-sectional study of Medicaid enrollees in West Virginia shed light on some of the implications of opioid prescription duration limit policies. The findings suggest that opioid prescription duration limits tailored to different clinical settings are associated with reduced length of prescriptions for opioid-naive patients. Given concerns about the risk-benefit profile of restrictive opioid limits, the CDC recently proposed changes to its clinical guidelines, rolling back opioid prescription duration limits recommended in prior editions.^29^ With states and payers likely to codify those guidelines into policy, the need for research on the broader implications—intended and unintended—of these policies is both critical and time sensitive.

## Notes

Supplement 1. **eTable 1.** Opioid Prescription Duration Limits by State **eTable 2.** Full Model Output from Interrupted Time Series Model

Supplement 2.
Data Sharing Statement
