# Omission in the Funding/Support Section

**DOI:** 10.1001/jamahealthforum.2024.0060

**Published:** 2024-02-16

In the Original Investigation titled “Opioid Prescribing Patterns After Imposition of Setting-Specific Limits on Prescription Duration,”^1^ published on January 19, 2024, a grant number was omitted from the Funding/Support section. The complete funding information is as follows: “This work was funded by grant R21DA050047 from the NIDA (Allen, Pollini, Vaglienti, and Powell) and the Rand Opioid Tools and Information Center (Allen and Powell), which received grant P50DA046351 from the NIDA.” This article has been corrected.
