# Supplementary material for: Opioid Prescribing Patterns After Imposition of Setting-Specific Limits on Prescription Duration
Source: JAMA Health Forum. 2024 Jan 19;5(1):e234731. doi: 10.1001/jamahealthforum.2023.4731 (PMC10799257; doi:10.1001/jamahealthforum.2023.4731)
Supplement: Supplement 1. — eTable 1. Opioid Prescription Duration Limits by State eTable 2. Full Model Output from Interrupted Time Series Model [file jamahealthforum-e234731-s001.pdf]

Supplemental Online Content

Allen LD, Pollini RA, Vaglienti R, Powell D. Opioid prescribing patterns after imposition of setting-specific limits on prescription duration. *JAMA Health Forum*. 2024;5(1):e234731. doi:10.1001/jamahealthforum.2023.4731

eTable 1. Opioid Prescription Duration Limits by State

eTable 2. Full Model Output from Interrupted Time Series Model

This supplemental material has been provided by the authors to give readers additional information about their work.

eTable 1. Opioid Prescription Duration Limits by State

Policy	# of States	List of States
3 Day Limit	2	Florida, Kentucky
5 Day Limit	3	Arizona, North Carolina, New Jersey
7 Day Limit	24	Arkansas, Colorado, Connecticut, Delaware, Hawaii, Indiana, Louisiana, Massachusetts, Maine, Michigan, Minnesota, Missouri, Montana, Nebraska, New Hampshire, New York, Ohio, Oklahoma, Pennsylvania, South Carolina, Utah, Virginia, Washington, Wyoming
10 Day Limit	2	Mississippi, Texas
14 Day Limit	1	Nevada
30 Day Limit	2	Illinois, Tennessee
20 doses	1	Rhode Island
Varies	3	Maryland, Vermont, West Virginia
**Source:** Lieberman A, Davis C. "Laws Limiting the Prescribing or Dispensing of Opioids" (The Network for Public Health Law web page.). https://www.networkforphl.org/resources/laws-limiting-the-prescribing-or-dispensing-of-opioids/		

eTable 2. Full Model Output from Interrupted Time Series Model

	Over Limit			Days Supply			Daily MME			>90 MME			Second Rx in 30 Days		
	**ED**	**Outpatient**	**Minor**	**ED**	**Outpatient**	**Minor**	**ED**	**Outpatient**	**Minor**	**ED**	**Outpatient**	**Minor**	**ED**	**Outpatient**	**Minor**
**Slope**	-0.00199*	-0.00108*	-0.0117***	-0.0142**	-0.0207**	-0.0602***	-0.102**	-0.00882	-0.103*	0.0000610	0.000675***	-0.0000664	-0.00222*	-0.00124*	-0.00107
	(0.000934)	(0.000477)	(0.00158)	(0.00499)	(0.00721)	(0.00866)	(0.0378)	(0.0286)	(0.0487)	(0.000194)	(0.000176)	(0.000189)	(0.000976)	(0.000534)	(0.000679)
**Level Shift**	-0.0703***	-0.0883***	-0.128***	-0.0975	-0.829***	-0.419***	0.417	0.103	2.168**	-0.00154	-0.0107***	0.0000260	-0.00215	-0.0191*	-0.00648
	(0.0171)	(0.00814)	(0.0231)	(0.0912)	(0.123)	(0.127)	(0.692)	(0.488)	(0.713)	(0.00355)	(0.00300)	(0.00277)	(0.0179)	(0.00911)	(0.00994)
**Slope Shift**	0.00322	0.000574	0.00440	0.00725	0.0110	0.0416**	-0.0261	-0.247***	-0.391***	-0.000313	-0.000955**	-0.000161	0.00273	0.000385	0.000882
	(0.00172)	(0.000811)	(0.00237)	(0.00921)	(0.0123)	(0.0130)	(0.0698)	(0.0486)	(0.0732)	(0.000358)	(0.000299)	(0.000284)	(0.00180)	(0.000908)	(0.00102)
**Constant**	0.203***	0.164***	0.590***	3.452***	5.609***	4.673***	30.19***	37.06***	35.19***	0.00628***	0.0108***	0.00467*	0.204***	0.173***	0.0572***
	(0.00859)	(0.00454)	(0.0165)	(0.0459)	(0.0686)	(0.0903)	(0.348)	(0.272)	(0.508)	(0.00179)	(0.00167)	(0.00197)	(0.00898)	(0.00508)	(0.00709)
**Observations**	9688	29146	5880	9688	29146	5880	9688	29146	5880	9688	29146	5880	9688	29146	5880
