# Supplementary material for: Opioid Prescribing Patterns After Imposition of Setting-Specific Limits on Prescription Duration
Source: JAMA Health Forum. 2024 Jan 19;5(1):e234731. doi: 10.1001/jamahealthforum.2023.4731 (PMC10799257; doi:10.1001/jamahealthforum.2023.4731)
Supplement: Supplement 2. — Data Sharing Statement [file jamahealthforum-e234731-s002.pdf]

Data Sharing Statement

Allen. Opioid Prescribing Patterns After Imposition of Setting-Specific Limits on Prescription Duration. *JAMA Health Forum*. Published January 19, 2024.
doi:10.1001/jamahealthforum.2023.4731

Data
Data available: No

Additional Information
Explanation for why data not available: As part of our data use agreement with West Virginia's Department of Health and Human Resources, we are not allowed to make individual patient data available to the public.
